# The Impact of Sugar Source on the Relationships Between Free Sugars Intake and Health: A Secondary Analysis

**DOI:** 10.3390/nu18091323

**Published:** 2026-04-22

**Authors:** Jennifer A. Peregoy, Laura Chiavaroli, John L. Sievenpiper, Stephen A. Fleming

**Affiliations:** 1 Traverse Science, Inc., Mundelein, IL 60060, USA; jennifer@traversescience.com; 2 Department of Nutritional Sciences, Temerty Faculty of Medicine, University of Toronto, Toronto, ON M5S 1A8, Canada; laura.chiavaroli@utoronto.ca (L.C.); john.sievenpiper@utoronto.ca (J.L.S.); 3 Toronto 3D Knowledge Synthesis and Clinical Trials Unit, Clinical Nutrition and Risk Factor Modification Centre, St. Michael’s Hospital, Toronto, ON M5B 1W8, Canada; 4 Li Ka Shing Knowledge Institute, St. Michael’s Hospital, Toronto, ON M5B 1W8, Canada; 5 Division of Endocrinology and Metabolism, Department of Medicine, St. Michael’s Hospital, Toronto, ON M5B 1W8, Canada; 6 Department of Medicine, Temerty Faculty of Medicine, University of Toronto, Toronto, ON M5S 1W8, Canada

**Keywords:** sugar-sweetened beverages, policy, regulations, public health, lipids, cardiovascular, glycemia

## Abstract

Background/Objectives: This secondary and exploratory meta-analysis re-evaluated 30 randomized controlled trials on free and added sugars (FS) detailed in the European Food Safety Authority’s (EFSA) report on the tolerable upper intake level for dietary sugars, focusing on the influence of food source (beverages, foods, or mixed) on cardiometabolic and anthropometric health. Methods: The EFSA’s method of analyzing the relative FS intake (difference between treatment and comparator arms, Δ%E^fs^) was used, with further adjustment for the reported intake of all sources of FS and energy. The EFSA’s “high vs. low” random-effects meta-analysis comparing groups with the highest and lowest FS intake was replicated, and additional exploratory dose–response meta-regressions (linear and non-linear) were performed, stratified by food source. Given the secondary and observational nature of the analysis, all source-stratified findings should be interpreted as hypothesis-generating, rather than causal. Results: There were no interactions between Δ%E^fs^ and food source for any outcome, and within a source there were linearly positive and statistically significant regressions for body weight (mixed), low-density lipoprotein cholesterol (LDL-C, foods), and uric acid (beverages). Across 13 outcomes, Δ%E^fs^ was positively and linearly related to greater fasting glucose, high-density lipoprotein cholesterol (HDL-C), and LDL-C, and non-linearly to body weight. However, the data were limited in their representation of FS intake at typical population levels, and there were insufficient data to investigate the effect of FS from foods on most anthropometric outcomes. Conclusions: Meta-regressive dose–responses revealed little relationship between Δ%E^fs^ from specific food sources and health outcomes, but such effects might be masked by confounding factors. Future trials that test realistic intakes of FS across diverse food matrices and account for dietary compensation would help to overcome limitations in the body of evidence.

## 1. Introduction

The value or threat of added and free sugars (FS) in the diet lacks scientific consensus. A 2022 Cochrane review of randomized controlled trials (RCTs) identified that added sugars have minimal-to-no effect on total cholesterol, low-density lipoprotein cholesterol (LDL-C), high-density lipoprotein cholesterol (HDL-C), triglycerides (TG), systolic and diastolic blood pressure, and fasting plasma glucose [1]. Conversely, a meta-analysis described in a report by the European Food Safety Authority (EFSA) on the tolerable upper intake level for dietary sugars identified a causal relationship with FS and risk of obesity and dyslipidemia from RCTs [2]. However, data from prospective cohorts did not suggest a positive relationship between intake of FS and risk of obesity or dyslipidemia [2]. The EFSA concluded that excess energy intake (rather than an effect specific to sugars themselves) and body weight gain appeared to be the main mechanism by which dietary sugars contribute to the development of chronic metabolic diseases in free living conditions.

Recent meta-analyses suggest that the food source (e.g., from beverages, juices, fruits, foods, etc.) of fructose-containing sugars mediates the relationship between sugar intake and adiposity [3], glycemic control [4] and other intermediate cardiometabolic outcomes [5,6,7] in RCTs and for cardiometabolic outcomes such as type 2 diabetes [8] and hypertension [9] in prospective cohort studies. The EFSA’s own high vs. low meta-analysis indicated the effect of higher FS intake on outcomes differed between FS from foods, beverages, or mixed sources, though the EFSA suggested the data were limited in this respect [2]. Further, a 2025 meta-analysis of cohort studies found sugar-sweetened beverages (SSB), but not added sugars (nor total sugars), were related to increased risk of type 2 diabetes [10]. Together, these findings suggest public health guidance may require more nuance than setting limits based on total exposures, and the risk of higher FS intake may depend on the food source.

The variable findings by regulators and academics indicate a challenge worthy of high-quality pursuit. Here, we aim to build upon a comprehensive systematic review described by the EFSA in their assessment of the tolerable upper intake level of FS.

### EFSA’s Findings and Select Limitations

From RCTs, the EFSA ultimately made the following conclusions on a positive and causal relationship between the intake of FS and risk of: obesity and dyslipidemia (moderate certainty); non-alcoholic fatty liver disease or non-alcoholic steatohepatitis (NAFLD/NASH) and type 2 diabetes (T2D, low certainty); and hypertension (low certainty). No RCTs were available to assess the risk of cardiovascular diseases [2]. One of many noted limitations of such findings was that the body of evidence was disproportionately composed of studies on SSB, while the EFSA estimated that the primary foods contributing to intake of FS sugars were “sugars and confectionery”, beverages (SSB, fruit and vegetable juices), and fine bakery wares (e.g., biscuits, buns, cakes, etc.) among adults in many European countries [2]. The EFSA estimated mean intakes of total FS ranged from 6 to 17% energy (E) and the 95th percentile intakes ranged from 13 to 35% E among adults from national surveys conducted across 19 European countries [2].

The objective of the present analysis was to address specific sources of uncertainty and limiting factors described by the EFSA related to the exposure calculation and food source of FS. Primarily, we set out to explore uncertainties related to the source (beverages, foods, or mixed sources), and the relative vs. absolute amounts of FS consumed. While there were numerous limitations described in the EFSA’s report, the following points summarize those we set out to address.

1. Methodological constraints
  a. Use of relative exposures
    i. For example, studies comparing groups consuming 5% and 10% E from FS (%*E^fs^*) were represented identically to those comparing 20% and 25% E, as the between-arm difference (∆%*E^fs^*) in both is 5%.
  b. Use of fractional exposures, not total FS
    i. “Between-arm differences in FS intake only refer to the dietary fraction that was manipulated by the intervention, and not necessarily to the intake of added and free sugars from all sources [i.e., including the background diet in addition to the intervention]…which was not always available.” [2]
    ii. Although pragmatic, this was problematic as it led to a mixture of studies where some manipulated the entire exposure to FS, whereas others manipulated a smaller portion.
  c. Comparator arms were set to a value of zero for FS intake, even when not true.
  d. Planned, but not actual, intakes were used.
    i. The target ∆%*E^fs^* to be administered, rather than the actual amount (adjusted for compliance and/or dietary compensation), was used.
    ii. Inability to address compliance to the planned administration.
2. Observed limitations in the body of evidence, available studies, and results.
  a. RCTs on SSB contributed a substantial amount of evidence for most outcomes other than lipids.
  b. Data were limited to explore whether the source of FS could be a modifying factor.
    i. Dose–response relationships (irrespective of food source) indicate ∆%*E^fs^* accounted for a small proportion of heterogeneity, suggesting most variation comes from some factor unrelated to FS intake (such as food source, between-study variability, methodological/population differences, study quality, etc.).

## 2. Materials and Methods

This study protocol was pre-registered with the Open Science Framework (OSF) [11] at https://doi.org/10.17605/OSF.IO/TJG7R.

### 2.1. Overall Approach

In contrast to the EFSA’s approach [2], which used the planned ∆%*E^fs^* of the manipulated portion of the diet (sections 1.1.2 and 1.6.7 of Annex L of the EFSA report), the present analysis evaluated both planned and actual intakes, exposure relative to the total diet and treatment fraction, and assessed the dose–response relationship between health outcomes and ∆%E^fs^ according to the food source (beverages, foods, or mixed sources).

### 2.2. Search, Screening, Eligibility

This study was a secondary analysis of the EFSA report [2], and therefore used the citations included in the EFSA report for in-scope outcomes. No additional searches or screening were conducted. Studies were limited to the RCTs used by the EFSA to answer their second subquestion, which assessed if the intake of FS was positively and causally associated with the risk of chronic metabolic diseases [2].

### 2.3. Data Extraction and Exposure Calculations

Data related to the study designs, subject characteristics, and outcomes were copied from the EFSA’s review, and the primary data extraction conducted was that related to the exposure to FS. Two reviewers independently extracted data from each study using pre-defined sheets in Microsoft Excel. Data were extracted as presented by study authors and transformed or reclassified as required for data analysis and visualization. Table 1 represents the multiple potential methods for exposure calculation. Where possible, total exposure to FS, adjusted for actual intake of all FS and reported total daily energy intake, was used. Where actual FS or energy intakes were not available, planned exposures were used. Exposure of comparator arms was not set to zero.

### 2.4. Data Synthesis

Data cleaning and transformation were performed in Microsoft Excel and RStudio (Posit PBC, Boston, MA, USA, Version 2024.12.0+467). Where necessary, medians and interquartile ranges (IQR), or alternative measures of variance were converted to means and standard errors. All units were converted to common units when possible.

### 2.5. Statistical Analysis

Analytic methods followed those detailed by the EFSA report as closely as possible. Details about the methods used by the EFSA for data standardization and analysis, and amendments for this analysis are provided in Table 2.

#### 2.5.1. High vs. Low Meta-Analysis

Given this is a high vs. low analysis comparing treatment groups (e.g., comparing the group consuming the most FS with those consuming the least, regardless of the amount), any deviations in exposure calculations conducted in the current assessment will have no impact on the pooled mean effects. The same methods described in Annex L of the EFSA report, and the same outcome data described in the main text of the EFSA report, were used [2]. Random-effects meta-analyses were conducted using Restricted Maximum Likelihood (REML) estimation, with pooled estimates weighted by the inverse variance method. A random effect for study was included to account for between-study variability. The results are presented as mean difference pooled effect estimates and 95% confidence intervals (CI). Between-study heterogeneity was assessed using Cochran’s Q test and the *I*^2^ statistic [12].

#### 2.5.2. Linear and Non-Linear Meta-Regression

Dose–response analysis was conducted with ∆%E^fs^ as the independent variable, effect size (difference in outcome between groups) as the dependent variable, and where possible, stratified by source (beverages [${∆\%\;E}_{beverages}^{fs}$], foods [${∆\%\;E}_{foods}^{fs}$], or mixed [${∆\%\;E}_{mixed}^{fs}$]) and source by ∆%*E^fs^* interaction as additional predictor variables. Meta-regression models were conducted for outcomes with ≥6 and stratified by source if there were ≥2 sources with ≥2 studies per source. For example, outcomes (e.g., body fat) nearly exclusively studied in a single source (e.g., beverages) could not be stratified. Where effects from the meta-regression are reported, values indicate the pooled effect of the slope of the relationship between exposure and outcome ± 95% CI.

Following the EFSA’s analytic approach, a restricted cubic spline model with three knots was used for non-linear regressions. Here, knot selection differed from the EFSA’s approach. The EFSA tested 8 models differing only by knot selection in their non-linear regression on fasting triglycerides, finding minimal-to-no effect of the knot selection [2]. In the present analysis, knots were positioned at 10, 15, and 21 ∆%*E^fs^*, corresponding to the median and interquartile range of the full dataset for ${∆\%\;E}_{total}^{fs}$ (∆%*E^fs^* unstratified by source), which are almost identical to the first model chosen by the EFSA (10, 15, and 20).

All meta-analytic procedures were conducted using RStudio (Posit PBC, Boston, MA, USA; Version 2024.12.0+467). Visualizations were created using RStudio and Tableau Cloud (Tableau Software, Mountain View, CA, USA; Server Version 2024.3.0).

## 3. Results

### 3.1. Body of Evidence

**Figure 1:**
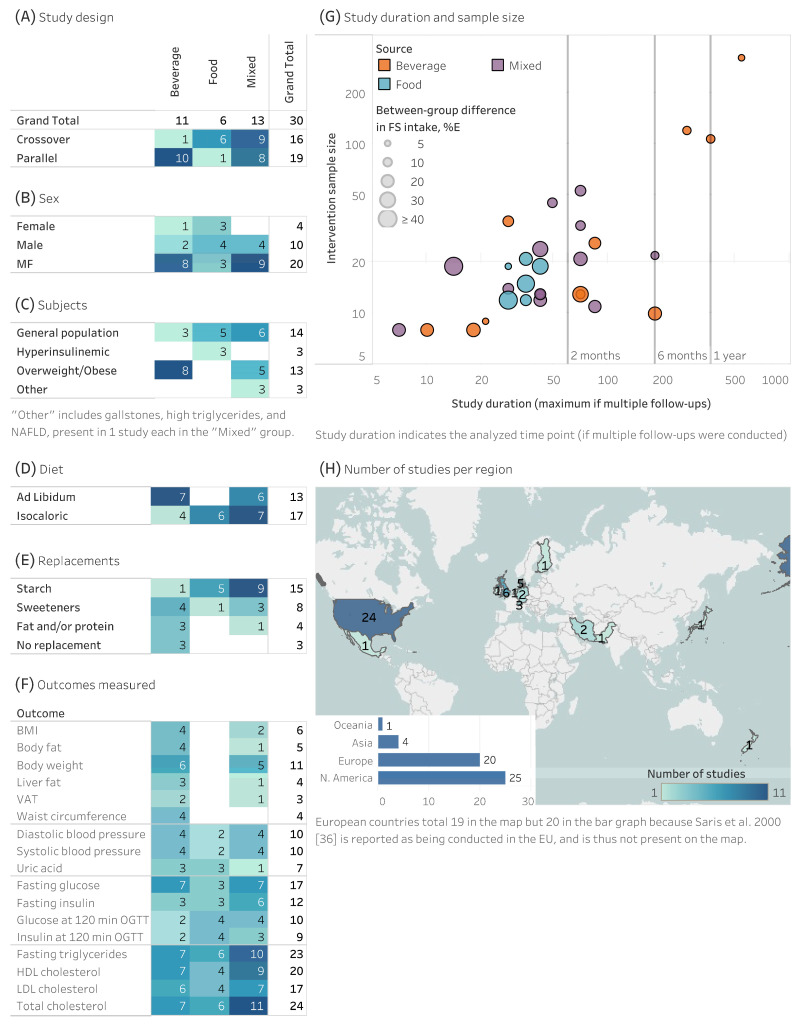
(**A**–**H**) Tables and figures represent the number of studies reporting a given study design element. Darker color intensity represents a higher number of studies. Panel (**G**) indicates the sample size of the intervention arm and the size of the bubbles indicate the difference in free sugar intake from the control group. Abbreviations: BMI, body mass index; MF, mixed-sex studies; NAFLD, non-alcoholic fatty liver disease; VAT, visceral adipose tissue; OGTT, oral glucose tolerance test; HDL, high-density lipoprotein; LDL, low-density lipoprotein; FS, free sugars; E, energy.

There were 30 studies available, the study characteristics of each are described in Supplementary Table S1 [13,14,15,16,17,18,19,20,21,22,23,24,25,26,27,28,29,30,31,32,33,34,35,36,37,38,39,40,41,42]. In general, studies were evenly designed as crossover or parallel trials (Figure 1A), were mixed sex or male-dominated (Figure 1B), were conducted on the general population or those with overweight/obesity (Figure 1C), used either ad libitum or isocaloric designs (Figure 1D), and usually swapped FS for starch or low/no-calorie sweeteners (Figure 1E). The most common outcomes measured were related to lipids (*n* = 17–24) and fasting glucose/insulin (*n* = 12–17), with fewer studies on blood pressure (*n* = 10), uric acid (*n* = 7), or anthropometric outcomes (*n* = 3–11) (Figure 1F). There were fewer than six studies for the following outcomes, which were not analyzed with meta-regression: liver fat, waist circumference, body fat %, and visceral adipose tissue. Most studies lasted less than 3 months in duration with sample sizes of the intervention groups between 10 and 50 (Figure 1G). The majority of studies were conducted in the United States and, to a lesser extent, in European countries (Figure 1H). There were no studies that directly compared food sources of FS (e.g., FS from foods vs. beverages) designed to directly assess the source; thus, all inferences on source are secondary and observational in nature.

Studies on foods and mixed sources of FS were similar to each other in that they more frequently replaced starch with FS, employed isocaloric crossover designs, and primarily enrolled participants from the general population (Figure 1A,C–E). Conversely, studies on beverages and mixed sources were similar, and distinct from those on foods, in their use of parallel design studies, longer duration studies, inclusion of mixed sex populations, recruitment of overweight and obese participants, use of ad libitum dietary protocols, and replacement of FS with sweeteners (Figure 1A–F).

Beverage studies (*n* = 11) were unique in that they included interventions adding FS without replacement (across 3 studies [20,21,29]) and exclusively focused on subjects without diagnosed disease or at-risk indicators. By comparison, food-based studies (*n* = 6) tended to be of shorter duration (4–6 weeks), with sample sizes from 12 to 21, primarily in general or hyperinsulinemic populations, strictly isocaloric, swapped FS with starch, and were the oldest studies conducted (5 studies between 1979 and 1989 [19,23,31,34,43]). Notably, no studies on foods reported anthropometric outcomes; these were primarily dominated by studies on beverages.

### 3.2. Exposures Used in Interventions

**Figure 2:**
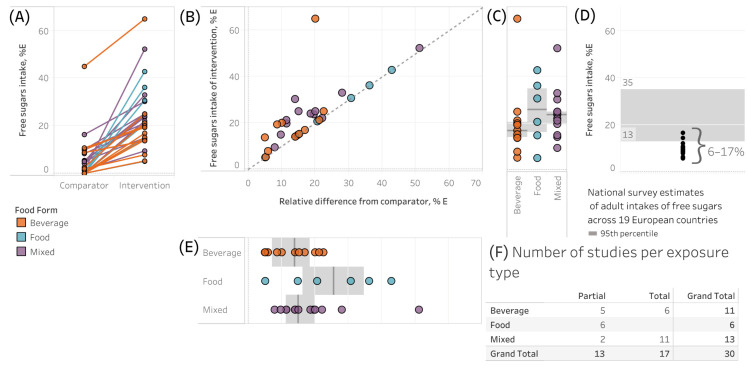
(**A**) Estimates of free sugar intake (%*E^fs^*) adjusted for all identifiable sources in comparator and intervention groups, adjusted for total energy intake, from the present analysis. (**B**) The relative difference (∆%*E^fs^*) between intervention and comparator groups (x-axis) plotted against the actual intake of the intervention (y-axis). Points along the dashed line indicate interventions compared to comparator groups at or near zero FS intake. (**C**) Intake of FS by food form. (**D**) EFSA’s estimates of the mean (6–17 %*E^fs^*) and 95th percentile for FS intake among 19 European countries. (**E**) The relative difference (∆%*E^fs^*) between interventions and comparator groups stratified by food form. (**F**) The number of studies by food form and if estimates represent partial or total estimates of FS from all sources. (**B**,**C**,**E**) Bands and shaded regions represent the median and interquartile range, respectively. Abbreviations: %E, percent energy; IQR, interquartile range.

Absolute FS intake of intervention groups ranged from 5.06 to 65 %E (Figure 2A). Seventeen studies compared these interventions with control groups also consuming FS, usually ≤16.3 %*E^fs^*, except for Thompson et al. [40], whose participants were assigned to consume unusually high intakes of FS in both the control and intervention groups (45–65 %E). The other 13 studies compared intervention groups to zero %*E^fs^* (or assumed to be zero/missing in cases where background dietary intake was not reported or calculable). Studies on the diagonal in Figure 2B represent intervention groups compared to comparators consuming near zero %*E^fs^*, whereas those not on the diagonal are comparisons to comparators with non-zero amounts of FS. The median ± IQR of the difference from comparator was 15.1 %*E^fs^* [10.3, 21.1], whereas the absolute intakes were 20.9 %*E^fs^* [14.8, 25.0] (Figure 2B).

Twenty studies were conducted at intake levels above the EFSA’s estimated mean exposure to %*E^fs^* across 19 EU countries (6–17 %*E^fs^*, Figure 2D) [13,14,15,18,20,23,25,26,27,28,30,31,32,34,39,40,41,42,43,44]; however, all but four [18,31,39,40] remained within the 95% percentile range (13–35 %*E^fs^*). %*E^fs^* and ∆%*E^fs^* were highest from foods and lowest from beverages (Figure 2C,E). Estimates including total %*E^fs^* were reported in 11/13 studies of mixed sources, in 6/11 of the beverage source studies, and 0/6 (completely absent) in studies of food sources (Figure 2F). When comparing partial and total estimates of %*E^fs^* were similar but generally lower when estimated from total sources for ∆%*E^fs^* (Supplementary Figure S1C,E).

Of 60 exposure estimates, 33% showed exact agreement between the EFSA’s analysis and the present analysis, with 67% differing (Supplementary Figure S2A,B). Rounding and unclear reasons each constituted 13% of the estimates (26% total), whereas those related to inaccuracies or adjustments for actual intakes were equally present at 20% each (40% total, Supplementary Figure S2A). Discrepancies related to rounding were minor (Supplementary Figure S2C). Those related to inaccuracies were larger and biased towards higher estimates in the present analysis, and those for unclear reasons or adjustments for real intake were variable but centered close to zero with minimal bias (Supplementary Figure S2C). The EFSA tended to underestimate the exposure for comparator groups, but there was no consistent bias (despite discrepancies) for intervention groups (Supplementary Figure S2D). Discrepancies by food form (Supplementary Figure S2E) and dietary portion (Supplementary Figure S2F) were both highly variable but centered near zero with minimal bias. In the present analysis, exposures were estimated from actual intakes in 18/30 studies, and planned intakes in 12/30.

### 3.3. Outcomes

**Figure 3:**
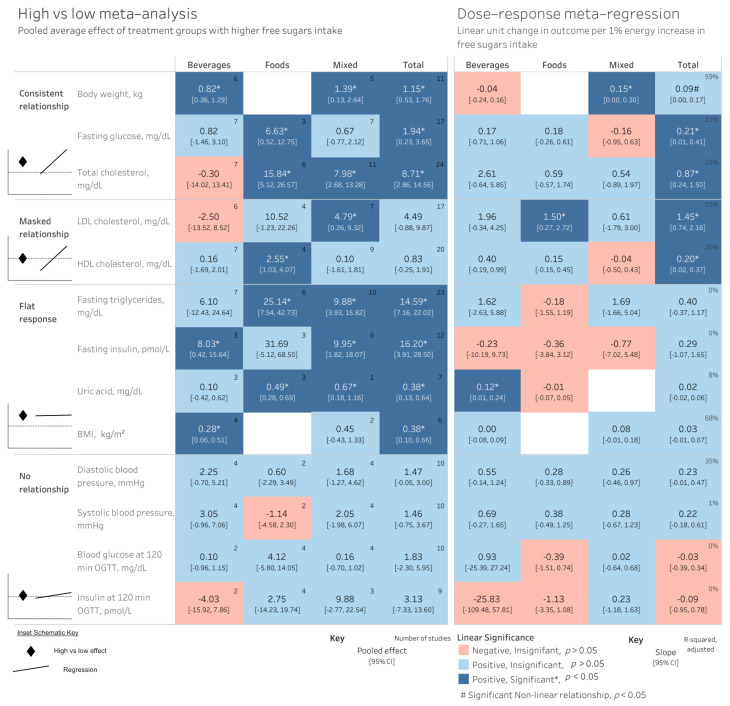
Comparison of results across EFSA’s high vs. low meta-analysis and the present linear and non-linear regressions, stratified by source. * Statistically significant effect at *p* < 0.05. ^#^ The shape of the curve was significantly non-linear at *p* < 0.05. Values represent the pooled estimate and 95% confidence interval in the high vs. low meta-analysis, and the estimate of the slope (with every 1% increase in % free sugars between groups) and confidence interval in the linear meta-regression. Inset schematic visually depicts the ways in which each analysis can vary between high vs. low and meta-regression analyses, with the dotted line indicating no effect of the treatment, and the diamonds and line indicated a high vs. low meta-analysis or meta-regression, respectively. The number of studies used for the high vs. low analysis is the same as that for the meta-regression. Abbreviations: LDL, low-density lipoprotein; HDL, high-density lipoprotein; BMI, body mass index; OGTT, oral glucose tolerance test.

The results from the meta-regression broadly fit within four patterns (Figure 3): (1) both the high vs. low analysis and meta-regression demonstrated an increase in the outcome (a consistent relationship) with higher FS intake, (2) the meta-regression found a relationship with ∆%*E^fs^* but the high vs. low analysis did not find a treatment effect of higher FS intake (a masked relationship), (3) the high vs. low meta-analysis found a treatment effect but there was no dose–response relationship (flat response), or (4) both analyses found no relationship with ∆%*E^fs^* (no relationship).

Fasting glucose and total, HDL and LDL cholesterol, and body weight (BW) (non-linearly) were each positively related with ${∆\%\;E}_{pooled}^{fs}$ but not other outcomes (Figure 3). By source, LDL-C was found to increase with ${∆\%\;E}_{foods}^{fs}$, body weight increased with ${∆\%\;E}_{mixed}^{fs}$, and uric acid increased with ${∆\%\;E}_{beverages}^{fs}$. Although for many outcomes the direction and/or magnitude of the relationship appeared to differ by source, there was no statistical interaction between source and ∆%*E^fs^* (all *p* > 0.05); thus, apparent differences by source should be interpreted cautiously. The relationship between outcomes and ${∆\%\;E}_{beverages}^{fs}$, however, was generally larger than other sources for all lipids, blood pressure, and post-prandial glucose/insulin outcomes. Among high vs. low analyses, effects (if statistically significant) were consistently weakest from beverages, and strongest from food (fasting glucose, total cholesterol, fasting insulin, fasting triglycerides, HDL-C, LDL-C) or mixed (body weight, BMI, uric acid) sources of FS. Given that the exposures in beverages were consistently the lowest (Figure 2C), it is unclear if such patterns can be attributed to the ∆%*E^fs^* or the source. Aside from body weight, there were no non-linear relationships identified.

#### 3.3.1. Outcomes Consistently Associated with Free Sugar Intake

**Figure 4:**
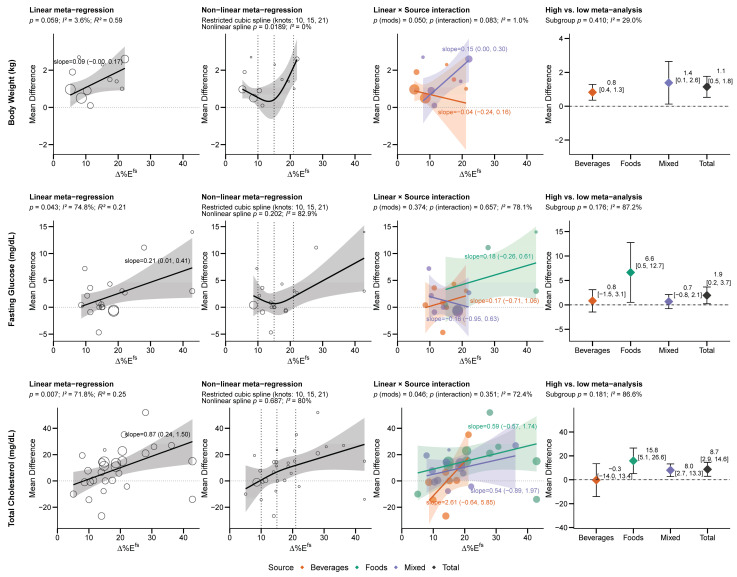
Outcomes consistently associated with increased free sugar intake across non/linear regressions and high vs. low analyses. Dotted lines indicate a mean difference of zero. Abbreviations: ∆%*E^fs^*, between-group difference in free sugar intake.

Body weight, fasting glucose, and total cholesterol were shown to increase with ${∆\%\;E}_{total}^{fs}$ in linear regressions (non-linear for body weight) and treatment groups in high vs. low analyses (Figure 4).

All studies found positive effect sizes for body weight with higher ∆%E^fs^, with linear regression indicating a positive relationship overall (0.09 kg per 1 ${∆\%\;E}_{total}^{fs}$, [0.00, 0.17], *R^2^* = 59%), statistically significant for mixed (0.15 kg per 1 ${∆\%\;E}_{mixed}^{fs}$ [0.00, 0.30]), but not for beverages (−0.04 kg per 1$\;{∆\%\;E}_{beverages}^{fs}$ [−0.24, 0.16]) (Figure 4). Non-linear regression revealed a slight J-shaped curve (*p* = 0.02), with increased BW at all ranges, but particularly above 16 ${∆\%\;E}_{total}^{fs}$. Body weight was the only non-linear relationship identified among all outcomes measured. Body weight had the highest R^2^ of all outcomes, with linear increases in ∆%*E^fs^* explaining 59% of the variability of the treatment effect.

Fasting glucose was linearly related with greater ${∆\%\;E}_{total}^{fs}$ (0.21 mg/dL per 1 ${∆\%\;E}_{total}^{fs}$ [0.01, 0.41], *R^2^* = 21%), but this was not evident in the regression by source, and non-linear regression indicated a near-significant relationship (*p* = 0.06). Though there was not a non-linear curve, visual inspection revealed effects were particularly small at <16 ∆%*E^fs^*, and coincidentally the higher doses were all conducted with food sources. High vs. low analysis indicated the strongest (and statistically significant) effects were found within food sources of FS and overall, but not within the beverages or mixed groups.

Each 1% increase in ${∆\%\;E}_{total}^{fs}$ increased total cholesterol by 0.87 mg/dL [0.24,1.50] (*R^2^* = 25%), though this did not reach significance within a given source, and the relationship was nearly completely linear. The high vs. low meta-analysis indicated total cholesterol increased with food, mixed, or all sources, but not beverages. Beverages tended to be assessed at lower ∆%*E^fs^*, which overlapped no effect <16 ∆%*E^fs^*, which may explain why the high vs. low pooled effect was near zero with high variability despite having the strongest positive linear relationship.

#### 3.3.2. Dose-Dependent Outcomes Masked by Minimal Treatment Effects

**Figure 5:**
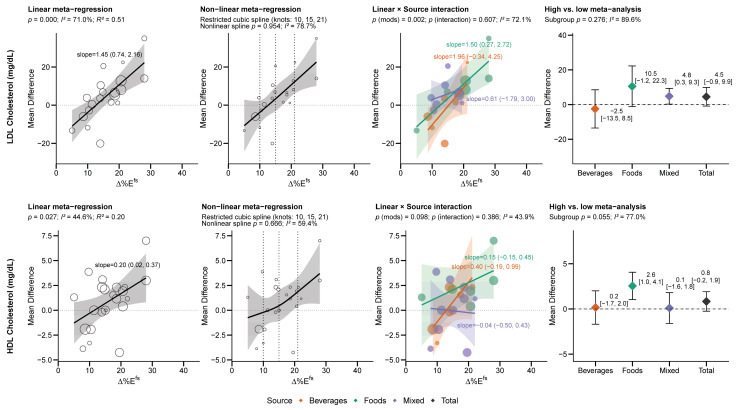
“Masked” effects wherein both LDL-C and HDL-C demonstrated linear relationships with increasing free sugar intake, but not in the high vs. low analysis. Dotted lines indicate a mean difference of zero. Abbreviations: ∆%*E^fs^*, between-group difference in free sugar intake.

${∆\%\;E}_{total}^{fs}$ was linearly related to increased LDL-C (1.45 mg/dL per 1 ${∆\%\;E}_{total}^{fs}$ [0.74, 2.16], *R^2^* = 51%), most strongly in beverages, though only statistically significant from food sources (Figure 5). LDL-C was one of the few variables with similar replication of studies across dose ranges and similar relationships across sources (Figure 5), indicating ∆%E^fs^ was consistently related to increased LDL-C regardless of the source. High vs. low analysis revealed the strongest effects from foods (though imprecise and not statistically significant) and mixed sources, but not beverages, and combined indicated no total effect.$\;{∆\%\;E}_{total}^{fs}$ was also linearly related to increased HDL-C (0.20 mg/dL per 1 ${∆\%\;E}_{total}^{fs}$ [0.02, 0.37], *R^2^* = 20%) (Figure 5). High vs. low analysis revealed no overall effect, though FS from foods and not mixed or beverage sources increased HDL-C. For both HDL-C and LDL-C, the confidence intervals exceeded zero at intakes >12–15 ${∆\%\;E}_{total}^{fs}$. This may explain why treatment effects were strongest in foods (which were studied under higher relative exposures) in the high vs. low analysis. Neither LDL-C nor HDL-C exhibited a non-linear relationship with ${∆\%\;E}_{total}^{fs}$.

#### 3.3.3. Outcomes with a Flat Response to Higher Free Sugars

Several outcomes were increased among treatment groups higher in %*E^fs^*, but not in a dose-dependent manner, hence exhibiting a “flat response” to ∆%*E^fs^*. These included fasting triglycerides, fasting insulin, uric acid, and BMI, which were all increased among treatment groups with greater %*E^fs^*. Although the *R^2^* (68%) was relatively higher for BMI, meta-regression indicated no statistically significant linear or non-linear relationships with ${∆\%\;E}_{pooled}^{fs}$ (Figure 3).

**Figure 6:**
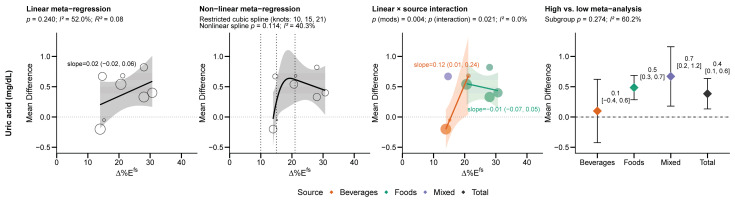
The relationship between exposure and uric acid was “flat”, as there was no dose–response relationship despite a positive pooled effect in the high vs. low analysis. Dotted lines indicate a mean difference of zero. Abbreviations: ∆%*E^fs^*, between-group difference in free sugar intake.

Both linear and non-linear models poorly fit uric acid and ${∆\%\;E}_{pooled}^{fs}$ (Figure 6), despite the visual appearance of both. However, ${∆\%\;E}_{beverages}^{fs}$ were statistically associated with increased uric acid (0.12 mg/dL per 1 ${∆\%\;E}_{beverages}^{fs}$ [0.01, 0.24]), and high vs. low random-effects estimates indicated an overall increase in uric acid, primarily in studies using foods and mixed sources, but not beverages. However, the exposure ranges for beverages and foods differed almost completely (Figure 6), making it impossible to determine whether the high vs. low effects represent a true dose–response or a source-specific difference.

#### 3.3.4. Outcomes Not Associated with Free Sugar Intake

Diastolic/systolic blood pressure and post-prandial blood glucose/insulin showed minimal-to-no relationship with ∆%*E^fs^* (Figure 3). The ∆%*E^fs^* from specific sources were not associated with any of the outcomes measured.

## 4. Discussion

We set out to build upon the EFSA’s analysis and explore specific limitations related to exposure calculations and the perceived over-representation of studies on SSB. Where possible, we estimated exposures on both an absolute (%*E^fs^* from all identifiable sources) and relative (∆%*E^fs^* between comparator and intervention groups) basis, using the actual (not planned) total energy and total FS intakes where possible. Although we found numerous discrepancies in the exposure calculations used by the EFSA, the most clearly identified systematic bias was an underestimate of the exposure of the comparator groups, given that the EFSA intentionally set the exposure of comparator arms to 0% even if they consumed FS [2]. Differences in exposure calculations, while sometimes large (e.g., >5% *E^fs^*), did not systematically differ across food sources of FS or across partial/total estimates of FS. While the EFSA was correct that studies on SSB contributed a substantial amount of evidence for many outcomes other than lipids (particularly anthropometrics), there were similar or greater numbers of studies in mixed formats. Beverages were particularly under-represented for glycemia-related outcomes like fasting insulin or oral glucose tolerance tests, and foods were generally fewer in number across the board.

Meta-regressive dose–responses revealed little relationship between ${∆\%\;E}_{total}^{fs}$ or specific sources and health outcomes, opposite to that of our expectations. The relationship with ${∆\%\;E}_{total}^{fs}$ was consistent for fasting glucose, total cholesterol, and body weight across high vs. low and meta-regression analyses; present in meta-regression but masked in the high vs. low analysis for HDL-C and LDL-C; absent in the meta-regression but present in the high vs. low analysis for body weight, BMI, fasting insulin, fasting triglycerides, and uric acid; and absent in both analyses for blood pressure, and post-prandial blood glucose/insulin. The only source-specific relationships observed were increases in uric acid with $∆{\%\;E}_{beverages}^{fs}$, increased body weight with ${∆\%\;E}_{mixed}^{fs}$, and increased LDL-C with ${∆\%\;E}_{foods}^{fs}$. However, the absence of a significant source-by-dose interaction term is a key inferential constraint on these findings. Given the exploratory and secondary nature of this analysis, all source-specific findings should be interpreted cautiously.

### 4.1. Caution on the Interpretation of Relative Exposures

The high vs. low meta-analysis is designed to assess the treatment effect of assigning one group to higher FS intake (i.e., “does the outcome change with higher FS intake?”), whereas the dose–response meta-regression assesses whether the magnitude of difference in FS intake between treatment groups (the relative intake, ∆%*E^fs^*) is related to the treatment response between studies (i.e., “Was the magnitude of the treatment effect related to the magnitude of increase in relative intake?”). A summary of the considerations for interpreting these results are listed in Box 1. We caution against the interpretation that the lack of a dose–response relationship illustrates proof that increased FS do not impact health. For outcomes with “flat” responses to ∆%*E^fs^*, such as uric acid (Figure 6), the interpretation is not necessarily that exposure and outcome are unrelated, but that the magnitude of the increase in exposure does not explain the variability in the response to the intervention.

Moreover, the exposure calculation used by the EFSA does not represent the total exposure of a treatment group to FS, only that of the difference between the intervention and comparator groups, sometimes only as it pertains to the manipulated portion of the diet, thus excluding exposure in the background diet. This is not an uncommon method, the 2019 Dietary Reference Intakes (DRI) report for sodium and potassium details a similar challenge [45]. The committee noted that virtually all eligible RCTs contributed only a single between-arm contrast in sodium intake, precluding characterization of absolute total intake across the dose–response range. Their solution was to combine multiple trials with different sodium intake levels for the control and intervention groups, the same process as was conducted here for FS. Further, they were able to use urinary sodium excretion as a biomarker of total dietary intake. Although similar biomarkers exist for total sugar intake [46], they do not exist for free or added sugars, which makes the partial-estimate problem in the present literature especially difficult to resolve. The DRI report further acknowledged that their meta-regression was ecologic in nature (performed at the study-level, not individual-level) and that substantial residual heterogeneity remained even after accounting for the sodium intake baseline outcomes. Despite these limitations, the committee considered characterization of the intake–response slope (rather than a precise absolute threshold) to be both meaningful and defensible. We adopt the same interpretive framework here: meta-regression of Δ%*E^fs^* is observational in nature and characterizes the direction and approximate magnitude of the dose–response relationship per unit of relative intake change but cannot be used to establish the absolute level of total FS intake at which health effects emerge. Future trials that measure and report total dietary exposure to FS from all sources, including the background diet, are needed before such thresholds can be reliably established.

**Figure 7:**
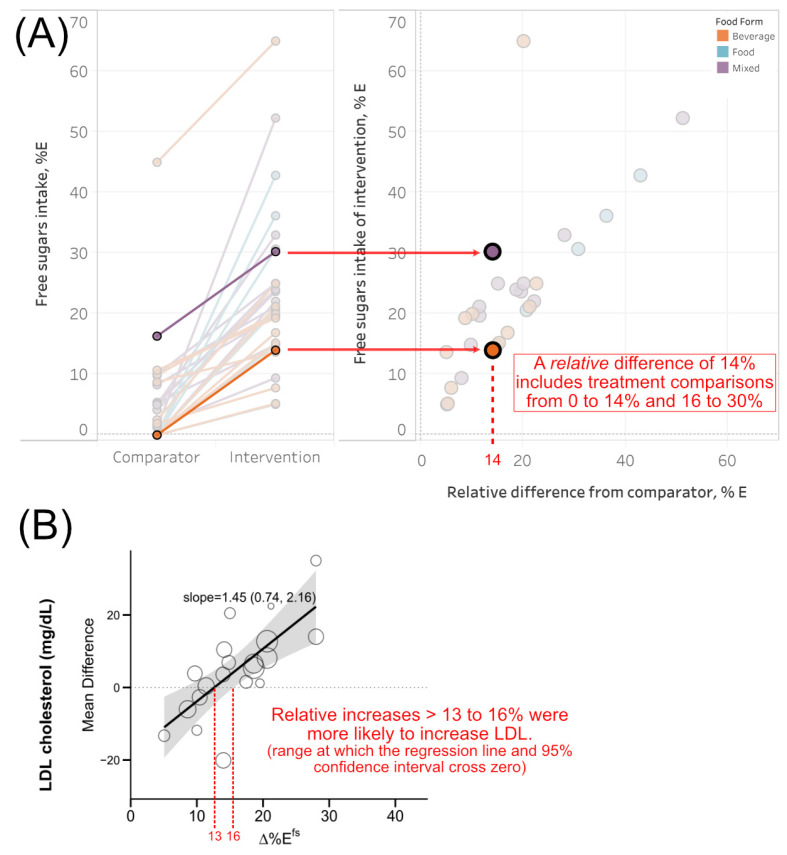
(**A**) Indicates how the same relative difference (14%) can translate to different absolute intakes. Here, the interventions used were ~30% and 14%, but the controls were reported to consume 16% and 0%, respectively. (**B**) Regressions indicate the relationship between increasing free sugar intake and LDL cholesterol, not the actual exposure to free sugars. Studies were more likely to report increases in LDL cholesterol if exposure was above 13 to 16% ∆%*E^fs^*.

The consequence of these limitations is illustrated in Figure 7A. For example, Majid et al. [29] and Lowndes et al. [27] studied groups consuming 0 and 14%*E^fs^*, or 16 and 30%*E^fs^*, yet both interventions are represented with the same ∆%*E^fs^* of 14% (Figure 7A). Across all studies, this disparity is evident by the interquartile range of ∆%*E^fs^* (10–21) representing higher actual intakes among interventions whose interquartile range includes 15–25%*E^fs^* (Figure 2B). The range of actual intakes is likely an underestimate given there were 13/30 studies with “partial” estimates where total FS intake could not be estimated (Figure 2F). Thus, this body of evidence cannot be used to quantify the true relationship between total FS intake and health, only relative increases from some sources.

Related to these constraints, the regression lines and confidence intervals for fasting glucose, total cholesterol, HDL-C, and LDL-C (Figure 4 and Figure 5) indicate modest or no effects below ~8–15 ∆%*E^fs^* (the point at which the regression line or confidence intervals exceed zero). Using LDL-C as an example (Figure 7B), studies tended to report increases more frequently above 13–16 ∆%*E^fs^*. To be clear, this does not establish 13–16 ∆%*E^fs^* as a definitive biological threshold for public health guidance, as it cannot be translated back into a threshold for total FS intake. Instead, this helps contextualize the finding that high vs. low analysis indicated no statistically significant effect for LDL-C despite the positive dose–response relationship. The high vs. low design tests whether studies conducted at higher %*E^fs^* produced larger mean effects than those conducted at lower intakes, which is only informative if the contrast between “high” and “low” study arms is sufficient to produce detectable differences in the outcome. This dataset suggests the contrast may need to exceed ~13–16 ∆%*E^fs^* for the effects on lipid fractions to emerge, yet the median exposure contrast across all included trials was 15 Δ*E*%*^fs^*, placing much of the evidence base at or below this range. This is a similar line of reasoning described by three studies by Lowndes et al. in this meta-analysis [25,26,27]: they found that %*E^fs^* at commonly consumed levels (8–30%) have modest-to-no effects across anthropometric, cardiovascular, and glycemia-related parameters [25,26,27]. Ultimately, a binary high vs. low comparison can yield attenuated or null pooled estimates even in the presence of a genuine underlying dose–response relationship, and the two methods of analysis are not necessarily discordant.

Box 1 **Summary of considerations for interpreting relative exposures.**

- A “flat” response may indicate the effect of increasing free sugar intake on health outcome (e.g., for fasting triglycerides/insulin/uric acid, and BMI) is not dependent on the magnitude of the increase.
- The use of relative exposures, the between-group difference in exposure, often fails to account for background dietary intake. This is a common method in nutrition science, but free sugars are particularly challenging due to the inability to estimate background dietary intake and control for it.
- High vs. low analyses can yield null results despite an existing dose–response relationship when the exposure range is centered around a null effect.

### 4.2. Energy Balance, Dietary Compensation, and Macronutrient Substitution

It is tempting to argue that changing %*E^fs^* is accompanied by other changes in energy and macronutrient intake, and those changes together are what compound to affect health. Previous reviews have identified that the effects of dietary sugars on body composition are mediated by isoenergetics [47,48]. The EFSA’s analysis of prospective cohorts indicated that adjustment for energy attenuates relationships between %*E^fs^* and health, but their subgroup analysis of RCTs with isocaloric designs (compared to ad libitum intakes) had stronger effects on fasting glucose/insulin, total cholesterol, LDL-C, HDL-C, and fasting triglycerides [2], consistent with an effect that is at least partially independent of energy intake. Yet, the present body of evidence is insufficient to simultaneously disentangle FS exposure from both energy intake and source (Box 2). A key confound is that foods were only studied under isocaloric designs (Figure 1D), whereas those in beverages and mixed formats were studied under either isocaloric or ad libitum designs.

There was also insufficient evidence to establish if changes in macronutrient intake act as a latent variable masking the effects observed. Neither the EFSA nor the present analysis assessed the impact of the treatments on macronutrient intake; however, our review indicated the available data for doing so were limited or that macronutrient intake was already controlled for in the design of the trials themselves. Fifteen studies were designed to replace starch or complex carbohydrates with FS while controlling for macronutrient intake [13,19,23,24,26,31,36,37,38,39,41,43], except for one study, whose design varied by 4% E protein between groups [18]. Of the eight studies that replaced FS with a sweetener, four did not report macronutrient intake [16,22,28,35], two found no differences in macronutrient intake [32,42], one kept macronutrient intake constant [17], and one found greater carbohydrate intake (but no change in fat or protein) with higher FS intake [14]. Of the three studies that made no explicit swaps with FS, two did not report macronutrient intake [20,29], and the other found no difference in energy or macronutrient intakes between groups [21]. This left four remaining studies that intentionally or effectively made isocaloric swaps of FS for protein and/or fat [25,27,30,40], too few to include as an adjusting factor for macronutrient intake given other potential confounds.

Taken together, the evidence suggests that where effects on cardiometabolic outcomes are observed in isocaloric trials, they are more plausibly attributable to the substitution of FS for starch than to changes in energy or macronutrient intake. Elsewhere, there were insufficient data to assess whether changes in macronutrient intake masked the effects found.

Box 2 **Relevance of energy and macronutrient intake to the results.**

- Energy and macronutrient intake were controlled for in the designs or effectively similar between groups in over half of the included studies. The remaining studies did not report energy or macronutrient intake.
- All studies on free sugars from foods were conducted with isocaloric designs, indicating the results in these studies cannot be attributed to dietary compensations in energy.

### 4.3. Limitations in Study Selection, Exposure, and Generalizability

Much of the above might be explained simply by artifacts of the study selection and resulting body of evidence (Box 3). Notably, this body of evidence is quite dated, with 10 of the 30 papers published before 1990 [18,19,22,23,31,33,34,39,40,42]; of these older studies, 5 comprise 83% of the 6 studies on food sources of FS [19,23,31,33,34], which also had higher doses than mixed and beverage sources. Broadly speaking, the exposures studied in the RCTs are unrealistic compared to real-life exposure to %*E^fs^*. Here, Figure 2C,D demonstrate that most exposures used in the studies were higher than the mean estimates of %E^fs^ in the EFSA’s analysis of nationally representative dietary intake survey data. Notably, four studies used sucrose interventions of 36–65% E [18,31,39,40], above even the 95th percentile intake across all 19 EFSA countries [2], yet produced surprisingly modest effects, likely introducing heterogeneity disproportionate to their small sample sizes (n = 6–19). It is also highly uncommon for populations to consume zero %*E^fs^* [2,49], and thus the artificial comparison of high FS intake to no FS intake is unrealistic.

Visualization of the study design elements revealed systematic differences in the studies used according to food source (Figure 1), particularly those from foods. RCTs on FS in foods were the only RCTs to study individuals living with hyperinsulinemia. Similarly, those from mixed sources included those with NAFLD, high triglycerides, or gallstones. Conversely, the studies on beverages primarily assessed subjects with overweight/obesity or the general population and included none of the subjects diagnosed with the aforementioned health conditions. All of the RCTs in foods were conducted between 4 and 6 weeks in duration, while those from other sources spanned longer durations (Figure 1). Studies in foods were exclusively conducted under isocaloric conditions, specifically with replacement of FS with starch (except for one case with sweeteners) (Figure 1). Conversely, studies on beverages rarely swapped FS with starch, and more frequently used sweeteners, other macronutrients, or made no swaps at all. The actual impact of these systematic biases in study design is unclear. However, it may have contributed to the pattern wherein effects were generally stronger with FS from foods in high vs. low analyses, but dose–response relationships were generally stronger among FS from beverages.

None of the included RCTs were designed to assess a dose–response relationship or the role of the food source, making the current meta-regression a secondary, observational analysis sensitive to confounds and latent variables [50]. Thus, it cannot be used for causal inference, and the surprising results are not necessarily in conflict with the results from the high vs. low meta-analysis. The meta-regressions do, however, provide insight into the potential relationship, strength, and predictive value of %*E^fs^* with health.

Perhaps the most likely explanation for the results is that the direction and magnitude of effects are plausible, yet the imprecision yielded statistically “insignificant” results. Meta-regression and subgroups both require greater power than a random effects meta-analysis [50], and this is compounded when meta-regressing by subgroups, especially in the presence of high imprecision. For example, LDL-C increased in all but one study assessing FS from foods, yet the high vs. low analysis for food sources of FS was insignificant (pooled effect of 10.52 mg/dL [−1.23, 22.26]) despite being the only subgroup with a statistically significant positive dose–response relationship between ${∆\%\;E}_{foods}^{fs}$ and LDL-C (1.50 mg/dL per 1%E [0.27, 2.72]) (Figure 3). Conversely, the pooled effect for mixed sources (a more precise estimate than from food sources) was significant despite being nearly half the magnitude (4.79 mg/dL [0.26,9.32]), yet it was found to have a modest and statistically insignificant linear relationship (0.61 mg/dL per 1 ${∆\%\;E}_{mixed}^{fs}$ [−1.76, 3.00]). If one assumes the direction and magnitude of the relationships are plausible and the imprecision is primarily an artifact of statistical power, then the dose–response slopes would suggest stronger relationships in beverages than mixed or food sources for most variables except fasting glucose and insulin. This would appear to conflict with the high vs. low effects, where effects were generally strongest among foods, but given the absence of a statistically significant source-by-intake interaction, neither interpretation can be advanced with confidence.

Box 3 **Methodological limitations of the included studies and meta-analysis.**

- The RCTs available manipulated exposures to free sugars that were uncommonly high compared to those described in national surveys and prospective cohorts.
- The durations, time of publication, populations recruited, and study designs were systematically different between studies on food, beverage, and mixed sources of free sugars.
- Systematic bias in study design and methodology might contribute to the heterogeneity of the results and why effects were generally stronger with free sugars from foods in high vs. low analyses, but dose–response relationships were generally stronger among free sugars from beverages.
- Imprecision and low statistical power likely masked the true relationship between the intake levels and their interaction with source.

### 4.4. Context with the Broader Evidence Landscape

Despite some reports that added sugars may have a modest effect on cardiometabolic health [1], the evidence that food source plays a moderating role (particularly in high vs. low meta-analyses) has been described by the EFSA and previous reviews [2,4,5,6,7,9,51]. What was unique in the present analysis was not only that there was a lack of interaction between exposure amount and food source, but that the relationship between health and ∆%*E^fs^* itself appeared modest. The dose-dependent and threshold-structured nature of added sugar and cardiovascular disease (CVD) associations in observational data provides further context for the effects observed here. Khan et al. [52] found that the associations of total sugars, fructose, and added sugars with CVD mortality in cohort studies followed non-linear dose–response relationships, with thresholds for harm emerging only above 133 g/day (26% E), 58 g/day (11% E), and 65 g/day (13% E), respectively. Below these thresholds, sugars were associated with neutral or even protective relationships with CVD mortality, and sucrose showed a linear inverse dose–response relationship across all intake levels. These threshold and direction patterns suggest that the exposure range captured by controlled feeding trials may critically affect the resulting null or attenuated findings among a high vs. low meta-analysis, as we argue in Section 4.1.

The contrast between RCT findings and prospective cohort evidence further contextualizes our results. Several large-scale meta-analyses of cohort studies have found that SSB intake (as a food category), but not FS (as a nutrient), is consistently and dose-dependently associated with cardiometabolic harm. An umbrella review of 73 meta-analyses by Huang et al. [53] builds upon this pattern. Across outcomes where both FS and SSB were examined, SSB were more consistently associated with adverse health than added sugars, suggesting the exposure construct may be a source of the discrepancy. Della Corte et al. [10] conducted a dose–response meta-analysis of prospective cohorts and found that each additional serving of SSB per day was associated with greater risk of type 2 diabetes, while added sugars showed no association with diabetes risk. Qin et al. [54] similarly found linear dose–response associations between SSB intake and risk of obesity, type 2 diabetes, hypertension, and all-cause mortality, with no evidence of non-linearity, and these associations were consistently larger in magnitude than any effects reported for FS in RCTs. Li et al. [55] extended this across a broader set of outcomes, with SSB associated with T2D, coronary heart disease, stroke, and all-cause mortality in dose–response analyses of prospective cohorts. Chen et al. [56] provide particularly compelling evidence on this point: in the Women’s Health Initiative cohort, consumption of regular soft drinks was associated with increased risk of peripheral arterial disease, whereas added sugar intake was not, with both exposures assessed in separate models adjusted for the same set of confounders including total energy intake. These data together suggest that food-level exposures appear to outperform nutrient-level exposures as predictors of health outcomes.

The conflation (or divergence) between sugary beverages and the analysis of FS as a nutrient is a limitation in the present analysis. The term “free sugars” is a relatively recent regulatory category [57] that most of the included RCTs predate and were not designed to study. Rather than manipulating exposure to FS as consumers encounter them across the diet, RCTs typically administered isolated mono- or disaccharides (e.g., fructose, sucrose, or glucose) at controlled doses as the intervention. Of the 30 studies included here, only 1 used language directly relating to free or added sugars (specifically, non-milk extrinsic sugars, NMES) [41], while others used terms such as “extrinsic sucrose” [37], “crystalline fructose” [38], or “added fructose” [58], which correspond to specific monosaccharide interventions rather than exposure to FS from the diverse dietary sources most consumers would encounter. As a result, the exposure endpoints in these RCTs are indirect and often incomplete proxies for FS intake as a dietary category. This is consistent with our previous scoping review demonstrating that RCTs tend to expose participants to artificially pure and high concentrations of individual sugars that are rarely reflected in observational dietary data [48].

We do not conclude from this that the RCT evidence is uninformative. RCTs remain well suited to establishing the directional effects of substituting one macronutrient or sugar source for another under controlled conditions, for example, whether replacing sucrose with starch improves lipid profiles, or whether sugar-sweetened beverages increase body weight relative to non-caloric alternatives. What the existing evidence cannot reliably support, given its reliance on isolated sugar exposures at artificial doses, is the establishment of precise dose–response thresholds for FS intake as a composite dietary construct. The discordant findings across reviews of this literature, including the limited and inconsistent relationships observed in the present analysis, may in part reflect this fundamental mismatch between the exposure construct used in regulatory guidance and the evidence available to support it. Future trials designed explicitly around FS as they are defined in the regulations, spanning diverse food sources, realistic intake levels, and adequate duration, would substantially strengthen the evidentiary basis for population-level dietary thresholds.

### 4.5. Recommendations for Future Research

According to the limitations previously described, we recommend that the research community consider the following to strengthen and build upon existing evidence:

1. Design head-to-head comparisons of FS intake from different food sources, across different intake ranges, in trials longer than 4–6 weeks.
2. Target realistic exposures to FS, both in quantity and in composition of the FS themselves.
3. Clearly and consistently report the total exposure to FS and potential dietary compensation, especially energy and macronutrient intake.
4. Greater investigation of the effects of FS on adiposity, including variables such as visceral adipose tissue, liver fat, body fat, and waist circumference.

## 5. Conclusions

This secondary analysis refines the EFSA’s assessment of dietary sugars by addressing limitations in exposure quantification and food source representation of FS. The primary finding is consistent with the EFSA’s in that higher between-arm FS intake was a weak or poor predictor of most cardiometabolic outcomes, with the exception of fasting glucose, total cholesterol, HDL-C, and LDL-C. The magnitude of observed effects was small or statistically insignificant at levels relevant to the typical population. Second, although there appear to be some source-specific moderating effects among the high vs. low and meta-regression analyses (e.g., specifically for body weight, uric acid, and LDL-C), there was not a statistical interaction between food source and the intake of FS and their impact on health; however, we caution that such effects could have been masked by confounding with some other factor (imbalance in number of studies on each source, variability of intake ranges, difference in study design, etc.). The use of relative, rather than absolute, differences in intake and lack of reported background dietary compensation contributed to heterogeneity and obscured the translation of findings to typical intake levels. Future research should (1) quantify total dietary exposure to FS from all sources, (2) assess dietary compensation effects and report energy and macronutrient intake, (3) compare food sources directly, and (4) employ intake levels reflecting real-world consumption patterns.

## Figures and Tables

**Table 1:** Example framework for quantification of exposures to added and free sugars.

		Absolute Free Sugar Intake, %E^fs^		Relative Free Sugar Intake, ∆%E^fs^	
Dietary Portion ^1^	Exposure Basis ^2^	Low Sugar Group	High Sugar Group	Difference in High vs. Low ^3^	Endpoint, ∆%E^fs^
Treatment	Planned	0%	5%	5%	Exposure typically used by the EFSA (5%), which the EFSA labelled the “Sugars dose [E%]”
Treatment	Actual	1%	4%	3%	
Entire diet	Planned	15%	20%	5%	
Entire diet	Actual	13%	22%	9%	Exposure used in current analysis (9%), labelled here as “∆%*E^fs^*”

^1^ In cases where changes to the entire diet were controlled, exposure from the treatment only and the entire diet are the same, and thus the dietary portion free sugars were estimated from is the “total” exposure. This differs from cases where only a fraction of the diet is manipulated and reported, where exposure is represented as a “partial” estimate (e.g., free sugars from beverages but not foods, or from either provided on an ad libitum basis). Where a fraction of the diet is manipulated, but the total exposure to free sugars from all sources is provided, the total exposure was used as the estimate in this analysis. The manipulated portion of free sugars was classified according to its source from foods, beverages, or mixed sources. Thus, studies that measured all possible sources of free sugars but only manipulated the beverage portion would be labelled with “beverage”. ^2^ Where possible, the actual exposure will be quantified and adjusted for both the actual total energy intake and total exposure to free sugars, whichever is available, and where actual intakes are not available, the planned value will be used as a default. ^3^ Data from intermediate groups in the case of multiple arms were not used by the EFSA, nor were they incorporated here.

**Table 2:** Protocol comparison between the EFSA report and present analysis.

Brief Description of EFSA’s Protocol ^1^	Amendments
1.1.1 Data standardization	
Estimation of free sugar intake on a % E basis using planned grams/day (g/d) intakes and total energy intake. Where energy intake is not available, imputations using 1800 kcals (females), 2000 kcals (males and females), or 2200 kcals (males) will be used. Estimates and interconversions mean and median values and variability parameters will be made to standardize data on a mean and standard error (SE) basis.	Where possible, actual energy intakes were quantified in addition to planned intakes. Extracted values were compared to the EFSA report for accuracy, and deviations described.
1.1.2. Mean effect computation	
Exposure estimation as the difference between groups in the manipulated fraction of free sugars between arms.	Exposure estimation was based on the actual and total identifiable exposure to free sugars, not solely that of the intended and manipulated fraction.
1.1.3 Data imputation	
Where sample size or variability is missing, the use of values from (1) the same arm at different time points, (2) the time point from different arms, or (3) the same arm and time point from a different study will be used.	No amendments
1.1.4 Correlation coefficient	
Use of correlation coefficient of 0.82 in calculations of the mean effect.	No amendments
1.4.1 Heterogeneity characterization	
Measurement using *I*^2^ value ^2^	No amendments
1.4.2 Pooled estimates (high vs. low analysis)	
Use of a random effects model with pooled estimates, using Restricted Maximum Likelihood (REML) to estimate variability.	No amendments
1.5-1.6 Meta-regressive dose–response analysis on a linear or non-linear basis	
A one-stage multivariate dose–response meta-analysis to investigate the relationship between sugar dose difference (E%) and fasting triglycerides (TG), fasting glucose, fasting insulin, body weight change, and uric acid.	Additional outcomes such as total cholesterol, high-density lipoprotein cholesterol (HDL-C), low-density lipoprotein cholesterol (LDL-C), body mass index (BMI), liver fat, visceral adipose tissue (VAT), waist circumference, diastolic/systolic blood pressure, and post-prandial blood glucose/insulin from oral glucose tolerance tests at minute 120. Any outcome where there were ≥6 studies was included.
Planned subgroup analyses	
Design, sex, diet (ad libitum, isocaloric with neutral/positive energy balance), source of sugars (foods, beverages, mixed), wash-out, run-in, duration, type of sugar (fructose glucose, mixed fructose and glucose), body weight effect change, and risk of bias were investigated visually (not in a meta-regression).	Subgroup meta-regression only with relation to the source of sugars (foods, beverages, mixed). Informal visual inspection of other subgroups was made only to identify potential confounding factors that would inform interpretation.

^1^ Unless otherwise specified, all procedures described in Annex L of the EFSA report were followed. ^2^ *I*^2^ quantifies the proportion of total variation in effect estimates across studies that is attributable to true heterogeneity rather than sampling error. It ranges from 0% to 100%, where 0% indicates no heterogeneity and higher values indicate increasing inconsistency across studies.

## Data Availability

All data are accessible in the Supplementary Materials provided.

## Supplementary Materials

The following supporting information can be downloaded at https://www.mdpi.com/article/10.3390/nu18091323/s1. Supplementary Table S1. Summary of studies included in the analysis. Supplementary Figure S1. (A) Estimates of free sugars intake (%*E^fs^*) adjusted for all identifiable sources in comparator and intervention groups, adjusted for total energy intake, from the present analysis. (B) The relative difference (∆%*E^fs^*) between intervention and comparator groups (x-axis) plotted against the actual intake of the intervention (y-axis). Points along the dashed line indicate interventions compared to comparator groups at or near zero free sugars intake. (C) Intake of free sugars by food form and whether the estimate was from a portion of the diet or all possible sources reported. (D) EFSA’s estimates of the mean (6–17%*E^fs^)* and 95^th^ percentile for free sugars intake among 19 European Union states. (E) ∆%*E^fs^* between interventions and comparator groups stratified by food form and dietary portion. (F) The number of studies according to food form of the free sugars exposure and if estimates represent partial or total estimates from all sources. BCDE) Bands and shaded regions represent the median and interquartile range, respectively. Supplementary Figure S2. (A) The frequency of discrepancies. (B) Comparisons between exposures calculated by EFSA and the present analysis. Points that deviate from the diagonal indicate different estimations. (C) Difference in estimates by reasoning, (D) treatment arm, (E) food form, and (F) portion of the diet that free sugars intake was estimated from. Shaded bands indicate the median and interquartile range. Positive differences indicate the secondary analysis estimate was higher than EFSA’s.
