# Supplementary material for: The Impact of Sugar Source on the Relationships Between Free Sugars Intake and Health: A Secondary Analysis
Source: Nutrients. 2026 Apr 22;18(9):1323. doi: 10.3390/nu18091323 (PMC13164700; doi:10.3390/nu18091323)
Supplement: Supplementary file 1 [file nutrients-18-01323-s001.zip › Peregoy 2026 Free sugars supplemental material.pdf]

Supplemental Material

The impact of sugar source on the relationships between free sugars intake and health

Jennifer A. Peregoy[1], Laura Chiavaroli[2,3,6], John L. Sievenpiper[2,3,4,5,6], and Stephen A. Fleming[1*]

[11]Traverse Science, Inc., Mundelein, IL, United States
[2]Department of Nutritional Sciences, Temerty Faculty of Medicine, University of Toronto, Toronto, Ontario, Canada
[3]Toronto 3D Knowledge Synthesis and Clinical Trials Unit, Clinical Nutrition and Risk Factor Modification Centre, St. Michael's Hospital, Toronto, Ontario, Canada
[4]Department of Medicine, Temerty Faculty of Medicine, University of Toronto, Toronto, Ontario, Canada
[5]Division of Endocrinology and Metabolism, Department of Medicine, St. Michael's Hospital, Toronto, Ontario, Canada
[6]Li Ka Shing Knowledge Institute, St. Michael's Hospital, Toronto, Ontario, Canada

Abstract

Background/Objectives: This secondary meta-analysis re-evaluated 30 randomized controlled trials on free and added sugars (FS) detailed in the European Food Safety Authority's (EFSA) report on the tolerable upper intake level for dietary sugars, focusing on the influence of food source (beverages, foods, or mixed) on cardiometabolic and anthropometric health. **Methods**: EFSA's method of analyzing the relative FS intake (difference between treatment and comparator arms, $\Delta\%E^{fs}$) was used, with further adjustment for the reported intake of all sources of FS and energy. EFSA's "high vs low" random-effects meta-analysis comparing groups with the highest and lowest FS intake was replicated, and additional dose-response meta-regressions (linear and non-linear) were performed, stratified by food source. **Results:** *There were no interactions between $\Delta\%E^{fs}$ and food source for any outcome, and within a source* there were linearly positive and *statistically significant regressions for body weight (mixed), LDL-C (foods), and uric acid (beverages). Across 13 outcomes, $\Delta\%E^{fs}$ was positively and linearly related with greater fasting glucose, HDL-C, LDL-C, and non-linearly with body weight. However, the data were limited in their representation of FS intake at typical population levels, and there was insufficient data to investigate the effect of FS from foods on most anthropometric outcomes.* **Conclusions**: Meta-regressive dose-responses revealed little relationship between $\Delta\%E^{fs}$ from specific food sources and health outcomes, but such effects might be masked by confounding factors. Future trials that test realistic intakes of FS across diverse food matrices and account for dietary compensation would help to overcome limitations in the body of evidence.

Supplemental Table S1. Summary of studies included in the analysis

First author, year	Study design, duration (weeks)	Age group	Comparisons	Food form	Sugar dose (%E)	Energy Balance	Dietary Design	Outcomes assessed
Black et al., 2006[13]	Crossover, 6	Adult	Sucrose levels	Mixed	10, 25	Isocaloric w/neutral energy balance	Controlled	Blood pressure, Glucose, Insulin, LDL-C, HDL-C, TC, TG
Campos et al., 2015[14]	Parallel, 12	Not reported	ASSD vs SSSD	Beverage	0, 18	NA	Ad libitum	BMI, Body fat, Body weight, Blood pressure, Glucose, Insulin, Liver fat, TC, TG, Uric acid, VAT, HDL-C
Despland et al., 2017[15]	Crossover, 1.14	Not reported	Starch vs. Honey vs. Glucose/Fructose	Mixed	0, 25, 25	Isocaloric w/neutral energy balance	Controlled	Glucose, Insulin
Ebbeling et al., 2012[16]	Parallel, 52	Adolescent	ASSD vs. SSSD, Fruit juice	Beverage	0, 17	NA	Ad libitum	BMI, Body weight
Gostner et al., 2005[17]	Crossover, 4	Adult	Isomalt vs Sucrose	Food	0, 6	Isocaloric w/neutral energy balance	Controlled	LDL-C, HDL-C, TG, TC
Groen et al., 1966[18]	Crossover, 5	Adult	Starch vs Sucrose	Food	0, 30	Isocaloric w/neutral energy balance	Controlled	TC
Hallfrisch et al., 1983a[19]	Crossover, 5	Adult	Starch vs. Low fructose vs. High fructose	Food	0, 7.5, 15	Isocaloric w/neutral energy balance	Controlled	Glucose, Blood pressure, Insulin, TG, LDL-C, TC, HDL-C
Hernandez-Cordero et al., 2014[20]	Parallel, 36	Adult	Water vs. SSB	Beverage	0, 20	NA	Ad libitum	BMI, Body weight, Body fat, Blood pressure, Glucose, TG,

								HDL-C, LDL-C, TC, Waist circumference
Hollis et al., 2009[21]	Parallel, 12	Adult	No beverage vs. Grape juice vs. Grape drink	Beverage	0, 18, 18	NA	Ad libitum	BMI, Body weight, Glucose, TG, LDL-C, TC, HDL-C
Huttunen et al., 1976[22]	Parallel, 72	Adolescent, Adult	Xylitol vs. Fructose vs. Sucrose	Mixed	0, 14, 16	NA	Ad libitum	Glucose, TG, TC, Uric acid
Israel et al., 1983[23]	Crossover, 6	Adult	Sucrose levels	Food	2, 15, 30	Isocaloric w/neutral energy balance	Controlled	Glucose, Blood pressure, Insulin, TG, LDL-C, TC, Uric acid, HDL-C
Lewis et al., 2013[24]	Crossover, 6	Adult	Sucrose levels	Mixed	5, 15	Isocaloric w/neutral energy balance	Controlled	Glucose, Blood pressure, Insulin, TG, LDL-C, TC, HDL-C
Lowndes et al., 2014a[26]	Parallel, 10	Adult	Sucrose levels vs HFCS levels	Beverage	10, 20	Isocaloric w/neutral energy balance	Partial control	HDL-C, Body fat, TG, LDL-C, TC, Waist circumference
Lowndes et al., 2014b[27]	Parallel, 10	Adult	Sucrose levels vs HFCS levels	Beverage	8, 18, 30	Isocaloric w/neutral energy balance	Controlled treatment	HDL-C, Body fat, Blood pressure, Glucose, TG, LDL-C, Liver fat, TC, Uric acid, Waist circumference
Lowndes et al., 2015[25]	Parallel, 10	Adult	Milk vs. Fructose vs. Glucose vs. Sucrose vs. HFCS	Beverage	0, 9, 18	Isocaloric w/neutral energy balance	Controlled treatment	Glucose, Insulin
Maersk et al., 2012[28]	Parallel, 24	Adult	Milk vs. Water vs. ASSD vs. SSSD	Beverage	0, 18	NA	Ad libitum	Glucose, Body weight, Blood pressure, Insulin, TG, LDL-C, Liver fat,

								TC, Uric acid, VAT, HDL-C
Majid et al., 2013[29]	Parallel, 4	Adult	No beverage vs. Honey	Beverage	0, 8	NA	Ad libitum	Glucose, TG, LDL-C, TC, HDL-C
Markey et al., 2016[30]	Crossover, 8	Adult	NMES levels	Mixed	6, 16	NA	Ad libitum	BMI, Body weight, Blood pressure, Glucose, Insulin, TG, LDL-C, TC
Moser et al., 1986[31]	Crossover, 4	Adult	Starch vs. Sucrose	Food	0, 43	Isocaloric w/neutral energy balance	Controlled	Glucose, Insulin, TG, TC
Raben et al., 2002[32]	Parallel, 10	Adult	Sweeteners vs. Sucrose	Mixed	0, 23	NA	Ad libitum	HDL-C, BMI, Body fat, Body weight, Blood pressure, Glucose, Insulin, TG, TC
Reiser et al., 1979[33]	Crossover, 6	Adult	Starch vs. Sucrose	Food	0, 30	Isocaloric w/neutral energy balance	Controlled	Glucose, TG, Insulin, TC, Uric acid
Reiser et al., 1989[34]	Crossover, 5	Adult	Starch vs. Fructose	Food	0, 20	Isocaloric w/neutral energy balance	Controlled	HDL-C, TG, LDL-C, TC, Uric acid
Ruyter et al., 2014[35]	Parallel, 72	Child	ASSD vs. SSSD	Beverage	0, 5	NA	Ad libitum	Body weight, Waist circumference
Saris et al., 2000[36]	Parallel, 24	Adult	High complex CHO vs. Control vs. High simple CHO	Mixed	19, 22, 38	NA	Ad libitum	HDL-C, Body weight, Glucose, Insulin, TG, LDL-C, TC
Smith et al., 1996[37]	Parallel, 24	Adult	Sugar-free vs. Sucrose	Mixed	0, 12	NA	Ad libitum	HDL-C, Body weight, TG, TC

Swanson et al., 1992[38]	Crossover, 4	Adult	Starch vs. Fructose	Mixed	0, 16.6	Isocaloric w/neutral energy balance	Controlled	Glucose, TG, LDL-C, TC, HDL-C
Szanto and Yudkin, 1969[39]	Crossover, 2	Adult	Starch vs. Sucrose	Mixed	0, 54	Isocaloric w/neutral energy balance	Partial control	Glucose, Insulin
Thompson et al., 1978[40]	Crossover, 1.43	Adult	Corn syrup levels vs. Sucrose levels	Beverage	45, 65	Isocaloric w/neutral energy balance	Controlled	Glucose, Insulin
Umpleby et al., 2017[41]	Crossover, 12	Adult	NMES levels	Mixed	6, 26	Isocaloric w/neutral energy balance	Partial control	HDL-C, Glucose, Insulin, TG, LDL-C, Liver fat, TC, VAT
Werner et al., 1984[42]	Crossover, 6	Adult	Sweeteners vs. Sucrose	Mixed	0, 24	NA	Partial control	HDL-C, Body weight, TG, LDL-C, TC

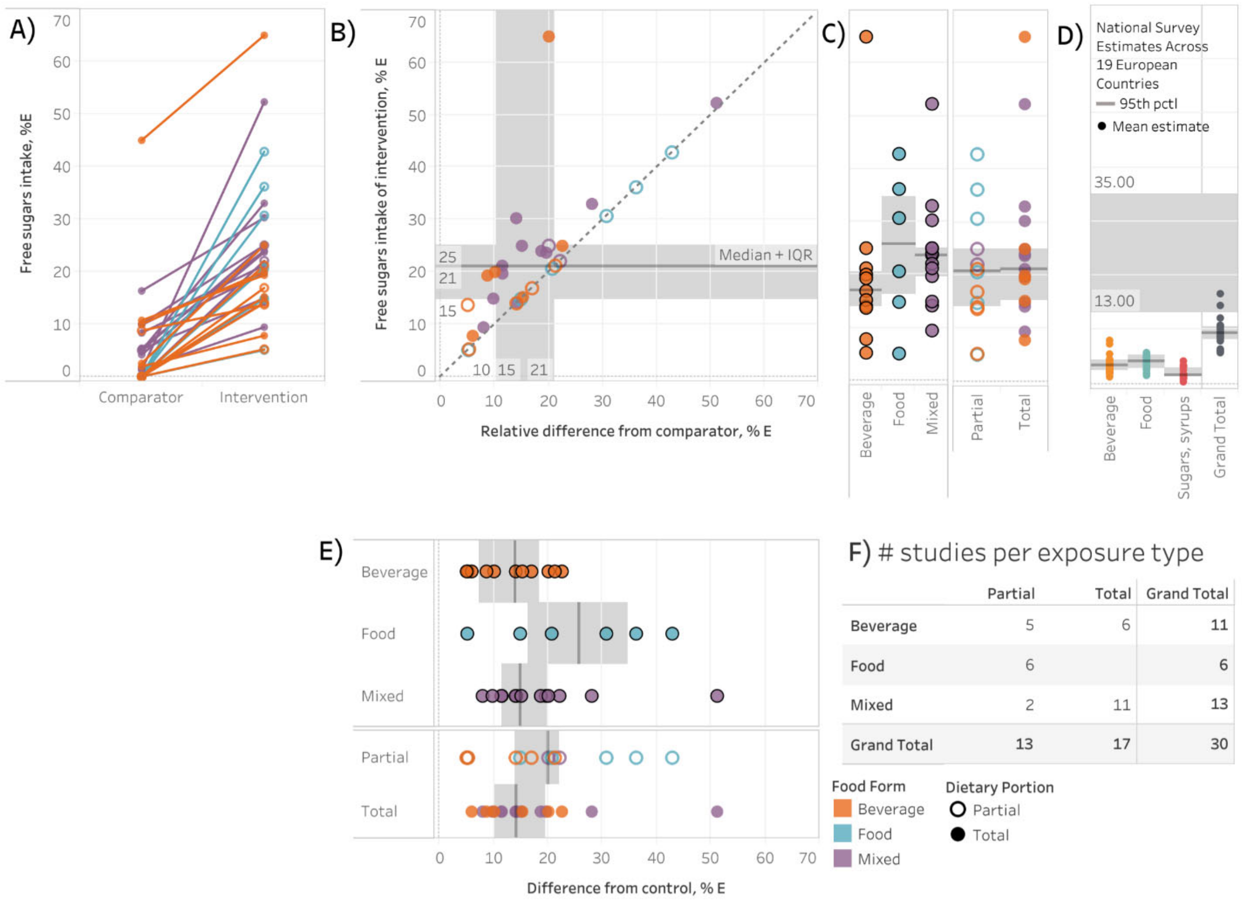

	Partial	Total	Grand Total
Beverage	5	6	11
Food	6		6
Mixed	2	11	13
Grand Total	13	17	30

Supplemental Figure S1. (A) Estimates of free sugars intake (%E^{fs}) adjusted for all identifiable sources in comparator and intervention groups, adjusted for total energy intake, from the present analysis. **(B)** The relative difference (Δ%E^{fs}) between intervention and comparator groups (x-axis) plotted against the actual intake of the intervention (y-axis). Points along the diagonal indicate interventions compared to comparator groups at or near zero free sugars intake. **(C)** Intake of free sugars by food form and whether the estimate was from a portion of the diet or all possible sources reported. **(D)** EFSA's estimates of the mean (6-17%E^{fs}) and 95th percentile for free sugars intake among 19 EU states. **(E)** The relative difference (Δ%E^{fs}) between interventions and comparator groups stratified by food form and dietary portion. **(F)** The number of studies according to food form of the free sugars exposure and if estimates represent partial or total estimates from all sources. **(BCDE)** Bands and shaded regions represent the median and interquartile range, respectively.

A) Frequency and reasoning of discrepancies

and % of instances a different estimate for free sugars intake was found among intervention and comparator groups between EFSA report and present secondary analysis

	None	Adjusted for real intake	Inaccuracies	Rounding	Unclear	Grand Total
Match	20 (33%)					20 (33%)
No match		12 (20%)	12 (20%)	8 (13%)	8 (13%)	40 (67%)
Grand Total	20 (33%)	12 (20%)	12 (20%)	8 (13%)	8 (13%)	60 (100%)

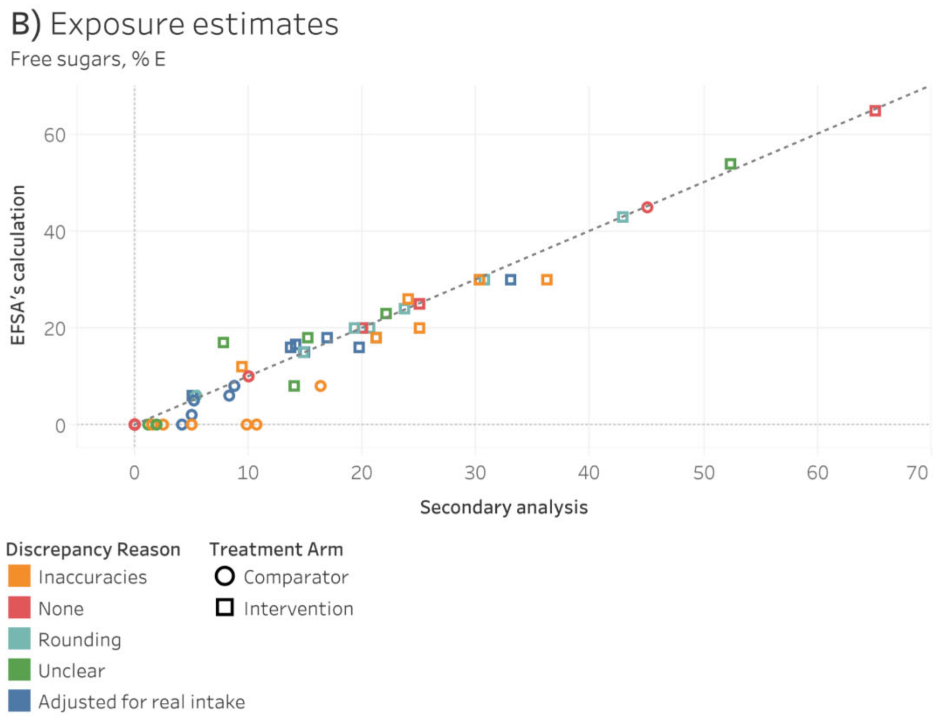

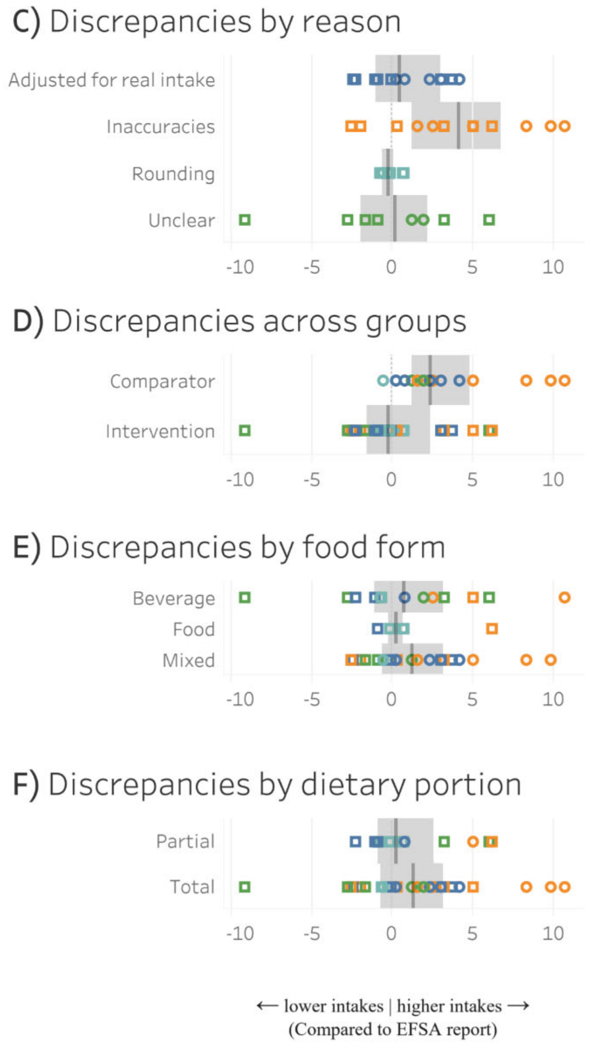

Supplemental Figure S2. (A) The frequency of discrepancies. **(B)** Comparisons between exposures calculated by EFSA and the present analysis. Points that deviate from the diagonal indicate different estimations. **(C)** Difference in estimates by reasoning, **D)** treatment arm, **(E)** food form, and **(F)** portion of the diet that free sugars intake was estimated from. Shaded bands indicate the median and interquartile range. Positive differences indicate the secondary analysis estimate was higher than EFSA's.
